# The human two-pore channel 1 is modulated by cytosolic and luminal calcium

**DOI:** 10.1038/srep43900

**Published:** 2017-03-02

**Authors:** Laura Lagostena, Margherita Festa, Michael Pusch, Armando Carpaneto

**Affiliations:** 1 Institute of Biophysics, National Research Council, Via De Marini 6, 16149 Genova, Italy; 2 Department of Biosciences, University of Milano, Via Celoria, 26, 20133 Milano, Italy

## Abstract

Two-pore channels (TPC) are intracellular endo-lysosomal proteins with only recently emerging roles in organellar signalling and involvement in severe human diseases. Here, we investigated the functional properties of human TPC1 expressed in TPC-free vacuoles from *Arabidopsis thaliana* cells. Large (20 pA/pF) TPC1 currents were elicited by cytosolic addition of the phosphoinositide phosphatidylinositol-(3,5)-bisphosphate (PI(3,5)P_2_) with an apparent binding constant of ~15 nM. The channel is voltage-dependent, activating at positive potentials with single exponential kinetics and currents are Na^+^ selective, with measurable but low permeability to Ca^2+^. Cytosolic Ca^2+^ modulated hTPC1 in dual way: low μM cytosolic Ca^2+^ increased activity by shifting the open probability towards negative voltages and by accelerating the time course of activation. This mechanism was well-described by an allosteric model. Higher levels of cytosolic Ca^2+^ induced a voltage-dependent decrease of the currents compatible with Ca^2+^ binding in the permeation pore. Conversely, an increase in luminal Ca^2+^ decreased hTPC1 activity. Our data point to a process in which Ca^2+^ permeation in hTPC1 has a positive feedback on channel activity while Na^+^ acts as a negative regulator. We speculate that the peculiar Ca^2+^ and Na^+^ dependence are key for the physiological roles of the channel in organellar homeostasis and signalling.

The endolysosomal system is composed of a series of internal compartments fundamental for cellular homeostasis and is involved in a variety of different physiological processes from signaling to cell growth up to defence mechanisms, to cite only few^1^,^2^. The family of two-pore channels (TPC) belongs to the group of endolysosomal membrane proteins and has two members in humans, namely TPC1, which is mainly expressed in endosomes, and TPC2, which mainly localizes to lysosomes^3^,^4^. From a structural point of view, TPC channels are homo-dimers^5^,^6^,^7^. The single monomer is formed by two shaker-type subunits covalently linked. Each shaker-type subunit has six transmembrane domains (S1 to S6) of which S4 contains a series of basic amino acids and is recognized to be the voltage sensor^6^, even though TPC2 is practically voltage-independent^8^,^9^,^10^,^11^. The regions between S5 and S6 (P-loops) form the selectivity filter of the permeation pore that confers cationic selectivity.

The physiological importance of TPC channels is underlined by recent findings of their involvement in different severe pathologies. TPC2 plays a role in neoangiogenesis processes linked to vascularization of solid tumors^12^, in neurodegenerative Parkinson disease^13^ and in Ebola virus infections^14^. In the heart, TPC1 is involved in the generation of Ca^2+^ waves after a period of ischemia and its deletion in mice is cardio protective^15^.

Despite the physiological relevance, the functional properties of TPC channels are not fully defined. The major problem in the functional characterization of these proteins is represented by their intracellular localization. This renders very difficult the application of electrophysiological techniques because of the sub-micrometric dimension of animal endosomes and lysosomes. Divergent results have been reported regarding the nicotinic acid adenine dinucleotide phosphate (NAADP)/PI(3,5)P_2_ sensitivity and the Ca^2+^ permeability of the channels. Using intracellular Ca^2+^ measurements^10^,^16^,^17^,^18^,^19^,^20^,^21^,^22^,^23^, redirection of the channels from lysosomes to the plasma membrane^24^, reconstitution in lipid bilayers^25^,^26^,^27^ and planar patch clamp of vacuolin enlarged lysosomes^10^,^14^,^28^,^29^, TPC1 and TPC2 have been reported to be activated by sub-micromolar concentrations of NAADP and to be highly permeable to Ca^2+^, leading to the suggestion that TPC channels represent the long sought for NAADP sensor^3^,^30^. In contrast, using classical patch clamp of enlarged lysosomes TPC1 and TPC2 were found to be insensitive to NAADP (even though in one study TPC2 could also be activated by NAADP^8^) and almost impermeable to Ca^2+^, but were instead activated by PI(3,5)P2 and highly permeable to Na^+^, and an indirect role in lysosomal Ca^2+^ release was suggested^11^,^31^,^32^.

An alternative approach to study mammalian lysosomal channels is to express them in plant protoplasts where they localize to the large lysosomal compartment, the vacuole, which is easy to isolate and amenable to classical patch clamp techniques^9^,^33^,^34^,^35^. Knocking out the single gene coding for the endogenous AtTPC1 the system is completely TPC-free^9^. Furthermore the vacuole has its cytosolic side facing the external bath solution, which is an ideal experimental situation to investigate cytosolic modulators^35^. Using this system we have previously found that hTPC2 is insensitive to NAADP, activated by PI(3,5)P2, and highly Na^+^ selective^9^, in agreement with Wang *et al*.^11^. Here, we focused our attention on human TPC1^15^,^26^,^27^,^32^,^36^,^37^, which is less characterized than TPC2. We found that the channel is dependent on PI(3,5)P2 but insensitive to NAADP. Currents are strongly voltage-dependent, mainly selective to Na^+^ but also Ca^2+^ permeable. Furthermore we found that both cytosolic and luminal Ca^2+^ are powerful modulators of hTPC1.

## Results

### hTPC1 channels is functionally expressed in *Arabidopsis thaliana* vacuoles

In mouse, two TPC1 isoforms have been found, the second one shorter than the first with an N-terminal deletion of 69 aminoacids^38^. Here we investigated a human isoform of TPC1 similar to the short mouse one.

**Figure 1:**
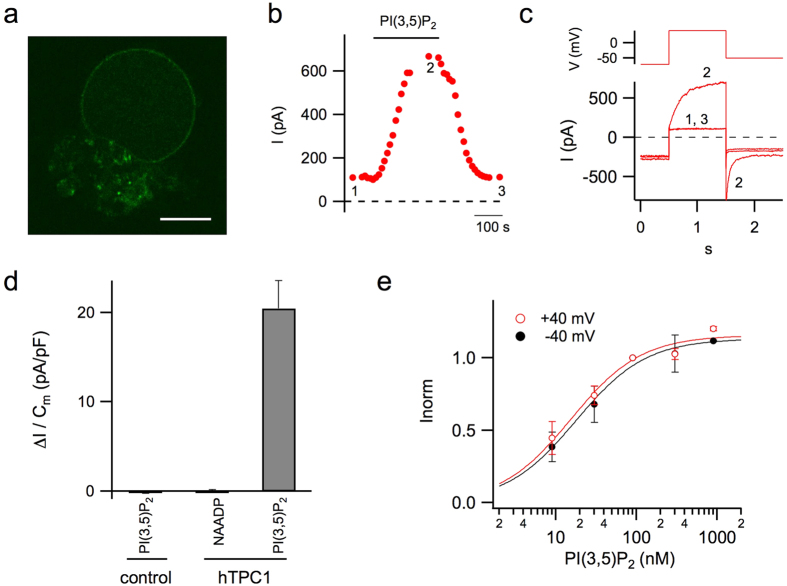
PI(3,5)P_2_ reversibly activated hTPC1 channel expressed in vacuoles isolated from Arabidopsis mesophyll cells. (**a**) Confocal image of a representative EGFP-positive vacuole showing expression of hTPC1 proteins on its membrane. Scale bar 10 μm. (**b**,**c**) Time course of current amplitude (**b**) recorded in response to bath application of 90 nM PI(3,5)P_2_; each point represents the steady-state current at +40 mV. The time course of current activation reflects the speed of solution exchange. Time points indicated by numbers correspond to the current traces, shown in C lower panel, in control (1), in the presence (2) and after wash out (3) of PI(3,5)P_2_. Currents were elicited by the voltage profile shown in c upper panel. (**d**) PI(3,5)P_2_ but not NAADP activates hTPC1 channels on Arabidopsis vacuoles. Summary plot of currents densities elicited at +40 mV by PI(3,5)P_2_ (90 nM, n = 57) or NAADP (100 nM, n = 6) on EGFP-tagged vacuoles (hTPC1). PI(3,5)P_2_ was ineffective when applied to untrasformed vacuoles (control, n = 53). (**e**) Dose-dependence activation of PI(3,5)P_2_-mediated current. For each vacuole, responses were normalized to the value obtained in the presence of 90 nM PI(3,5)P_2_ (n ranging from 3 to 6). Data were fitted with the Michaelis-Menten function I_max_/([PI(3,5)P_2_] +K_m_), with half-maximal activation (K_m_) of 15 ± 3 *v*M at +40 mV and 18 ± 3 *v*M at −40 mV. I_max_ was 1.16 ± 0.04 and 1.13 ± 0.03 respectively at + and −40 mV.

Human TPC1 (hTPC1) was transiently expressed in mesophyll protoplasts from the *Arabidopsis thaliana tpc1-2* mutant (AtTPC1 null background)^39^. Released EGFP-positive vacuoles (Fig. 1a) were viewed and selected under a fluorescence microscope to achieve patch-clamp recordings in the whole-vacuole configuration. Small (mostly background) currents were recorded in control conditions, whereas bath (equivalent to the cytosolic side) application of PI(3,5)P_2_ reversibly increased the current in response to a depolarizing step of +40 mV from a holding potential of −70 mV (Fig. 1b,c). The current gradually decayed to the basal level upon PI(3,5)P_2_ washout (Fig. 1b). PI(3,5)P_2_-evoked currents were observed in hTPC1-EGFP expressing vacuoles but not in untransformed control vacuoles (Fig. 1d), strongly suggesting that PI(3,5)P_2_-responses are due to activation of hTPC1 channels. As NAADP has been proposed to be a TPC-channel agonist^3^, we tested this intracellular calcium mobilizer on EGFP-positive vacuoles. Bath application of 100 nM NAADP on PI(3,5)P_2_ responding vacuoles did not elicit an increase of membrane current (Fig. 1d); these data point to a direct interaction of PI(3,5)P_2_ but not of NAADP with hTPC1.

The PI(3,5)P_2_-activated current increased with increasing PI(3,5)P_2_ concentration with a similar apparent binding constant at +40 mV and −40 mV, i.e. 15 and 18 nM (Fig. 1e), suggesting voltage-independent high affinity of PI(3,5)P_2_-interaction with hTPC1 channels.

### PI(3,5)P_2_-mediated current is voltage-dependent and largely carried by Na^+^ ions

**Figure 2:**
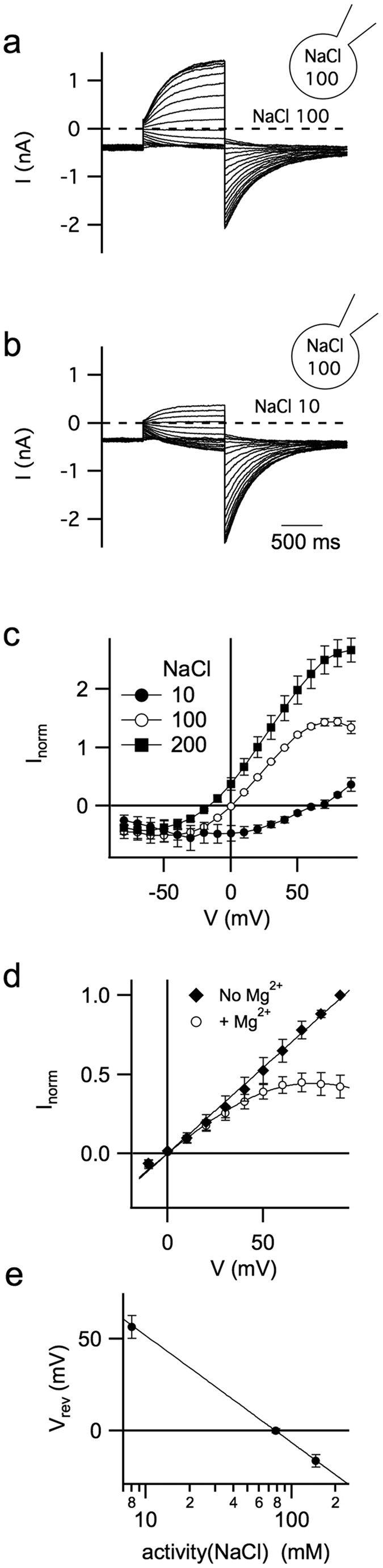
Human TPC1 is a voltage-dependent sodium channel. (**a**) Whole-vacuole currents recorded in the presence of 90 nM PI(3,5)P_2_ in standard symmetrical 100 mM-NaCl condition. MgCl_2_ 2 mM was present in the cytosolic bath solution. Currents were elicited by 1 sec-voltage pulses from −80 to +90 mV, in 10 mV-increments. Holding potential of −70 mV. (**b**) Same vacuole as in a, but bath (cytosolic) NaCl concentration was decreased to 10 mM (in the presence of 90 nM PI(3,5)P_2_). (**c**) Current–voltage relationships of PI(3,5)P_2_-evoked hTPC1 currents in the presence of 200 mM NaCl (n = 4), 100 mM (n = 8) or 10 mM (n = 4) in the bath. For each vacuole, current amplitudes evoked by 90 nM PI(3,5)P_2_ were normalized to the value at +40 mV in 100 mM NaCl-condition. (**d**) Effect of cytosolic magnesium on hTPC1 currents recorded at positive voltages. Currents were normalised to the value at +90 mV. We fitted the data, continuous lines, with the following equation: I_0_(V)/([Mg^2+^]/K_Mg_ exp(2δFV/RT)), where I_0_(V) was the linear fit of the current in the absence of cytosolic Mg^2+^. (**e**) Plot of reversal voltages versus cytosolic NaCl activity. Data were shown as mean ± standard deviation. The solid line represents the theoretical Nernst voltage for sodium.

To investigate the voltage-dependence of hTPC1-mediated current, EGPF-positive vacuoles were stimulated by increasing voltage pulses during bath perfusion of 90 nM PI(3,5)P_2_ (Fig. 2a). The response was larger at positive than at negative potentials indicating activation of a voltage dependent outward rectifying conductance. When cytosolic Na^+^ was diminished to 10 mM, outward currents were significantly reduced (Fig. 2b). Figure 2c summarizes the different current-voltage relationships of hTPC1 mediated currents when varying the cytosolic Na^+^ concentration; a large shift in the reversal voltage (V_rev_) from positive to negative values was recorded upon a cytosolic sodium concentration change from 10 to 200 mM. The full agreement of the experimental V_rev_ with the theoretical Nernst voltage for Na^+^ shown in Fig. 2e strongly points to hTPC1 as a Na^+^ permeable channel. In Fig. 2c the voltage dependent inhibition apparent at positive voltages in 100 mM sodium (the effect was less pronounced in 200 mM Na^+^) was due to the presence of 2 mM of cytosolic Mg^2+^: removing cytosolic Mg^2+^ eliminated the voltage dependent current inhibition (Fig. 2d). A similar effect has been reported for TPC2^8^. By using a Woodhull approach^40^, we estimated that Mg^2+^ binds to a site located along the permeation pore at an electrical distance (δ) from the cytosol of 0.36 ± 0.3 with an affinity constant at 0 mV (K_Mg_) of 20 ± 3 mM (see lines in Fig. 2d).

### Potassium and calcium selectivity of PI(3,5)P_2_-evoked current

**Figure 3:**
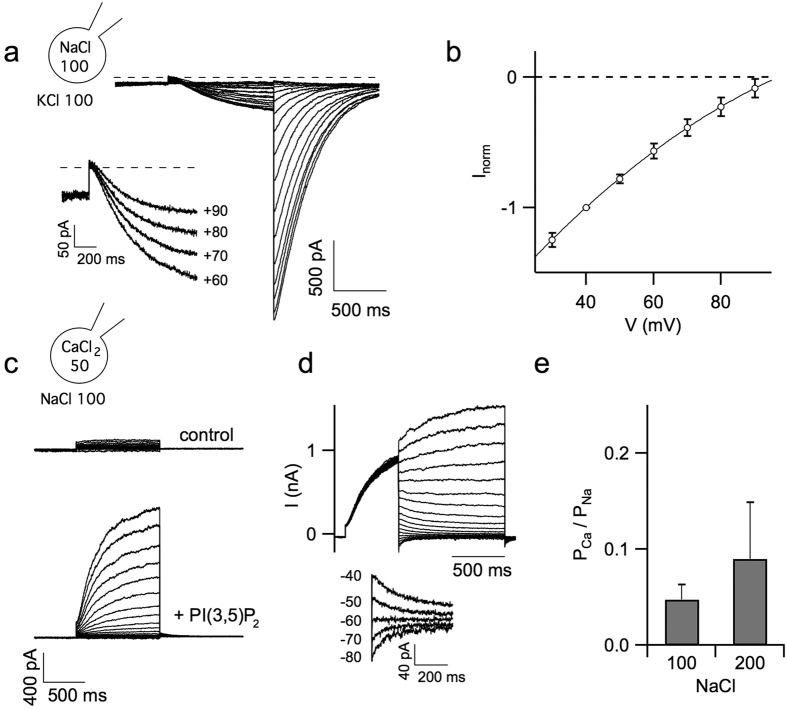
Potassium and calcium permeability of hTPC1 channels. (**a**) Representative traces of PI(3,5)P_2_-mediated currents recorded at different voltages from −80 to 90 mV, in 10 mV voltage step, after perfusion of 100 mM KCl (replacing 100 mM NaCl) in the bath solution. Holding potential of −70 mV, tail voltage of −50 mV. Inset: magnification of inward currents recorded from +60 to +90 mV. (**b**) Current-voltage relationship in the presence of 100 mM KCl (n = 6) in the bath solution. Current amplitudes were normalized to the value obtained at +40 mV. Dashed lines indicate the 0 level current. (**c**) Whole-vacuole recordings before (top) and after (bottom) PI(3,5)P_2_ bath perfusion. Pipette (luminal side) was filled with 50 mM CaCl_2_ in the place of 100 mM NaCl. Currents were recorded at voltages ranging to −90 to +80 mV in 10 mV step. Holding and tail voltage at −80 mV. (**d**) Tail currents recorded in response to potentials (1 sec-long) ranging from +70 mV to −100 in −10 mV decrement after 500 ms pre-pulse to +50 mV. Holding voltage: −80 mV. Inset: expanded view of tail currents recorded from −40 to −80 mV. (**e**) Permeability ratios, based on reversal voltage measurements (see Supplementary Fig. 2), in the presence of 100 mM (left, n = 11) or 200 mM (right, n = 4) cytosolic NaCl.

Replacing cytoplasmic Na^+^ by K^+^ in the presence of luminal Na^+^ resulted in a total disappearance of PI(3,5)P_2_ activated outward currents (Fig. 3a), while inward tail currents, reflecting Na^+^ flowing from lumen to cytosol, were still present, heavily suggesting a low hTPC1 permeability for K^+^. In this experimental condition the reversal voltage was larger than 90 mV as shown in the inset of Fig. 3a and in Fig. 3b pointing to a permeability ratio between K^+^ and Na^+^ lower than 2.8%. To measure the Ca^2+^ permeability, we substituted 100 mM Na^+^ with 50 mM Ca^2+^ in the pipette solution. In this bi-ionic condition large outward currents, reflecting Na^+^ flowing from the cytosol to the lumen, were present (Fig. 3c, *bottom*). To focus on Ca^2+^ permeability, tail currents were recorded by stepping from +70 to −100 mV (10 mV decrement), after a 500 ms activating pule to +50 mV (Fig. 3d, *top*). Exploring the responses on an expanded scale around the V_rev_, small inward tails were detectable. Ion permeability ratio between Ca^2+^ and Na^+^ (P_Ca_/P_Na_), measured by reversal potentials (Fig. 3d, *bottom*) was between 5 and 10% (Fig. 3e). It is worth noting that the reversal voltage was somewhat dependent on the applied protocol: the longer was the pre-pulse to +50 mV the more positive was V_rev_ (i.e. the higher was the apparent Ca^2+^ permeability). We attributed this effect to the entrance of Na^+^ into the lumen and mathematically corrected it (see Supplemental Fig. 1). These data indicate that Ca^2+^ can permeate hTPC1 although with a significantly lower permeability than Na^+^.

### Cytosolic calcium ions modulate PI(3,5)P_2_-activated currents

**Figure 4:**
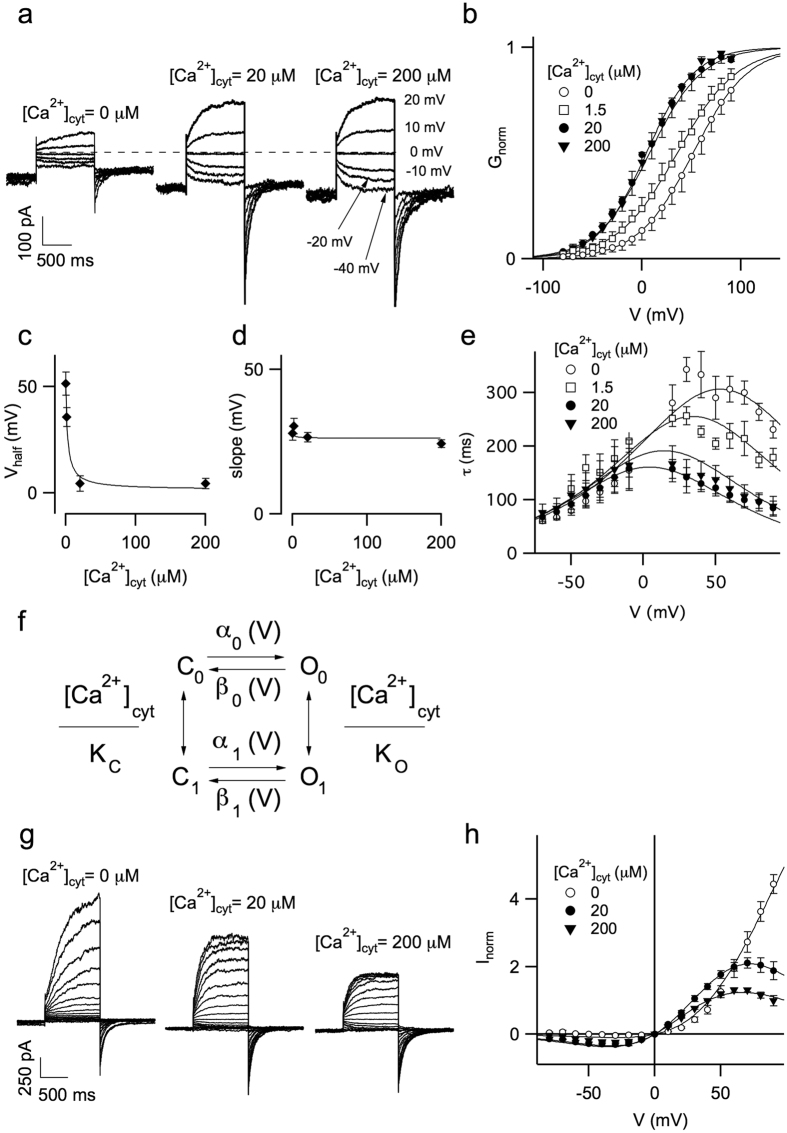
Cytosolic calcium modulated hTPC1 activity. (**a**) Recordings of hTPC1-mediated currents at different cytosolic Ca^2+^ concentrations. PI(3,5)P_2_ was present in the cytosolic bath solution at 90 nM. Voltages of the main pulse indicated in the left panel. Holding voltage: −70 mV, tail voltage: −50 mV. Standard (luminal) pipette solution with 1 mM CaCl_2_ added. (**b**) Normalized conductances (G_norm_) obtained from tail current peaks at −50 mV plotted as a function of the main voltage pulse at different cytosolic Ca^2+^ concentrations. Data from single vacuoles were fitted by Boltzmann function and normalized to the maximum value. Normalised conductances were the average of 4 different vacuoles. Continuous lines represented the Boltzmann fit. (**c**) Half voltage of activation of the Boltzmann fit shown in B versus cytosolic calcium concentration. (**d**) Slope of the Boltzmann fit shown in B versus cytosolic calcium concentration. Continuous lines in c and d were obtained by the scheme of (**f**) (see text and Supplemental Appendix). Data in **c** and **d** are shown as mean ± standard deviation. (**e**) Relaxation time constants versus applied voltage, at different cytosolic calcium concentration. Data from 4 different vacuoles. (**f**) Allosteric mathematical model describing modulation of hTPC1 by cytosolic Ca^2+^. (**g**) Same currents, as in A, adding missing traces elicited from −80 up to +90 mV. (**h**) IV relationships of stationary hTPC1 currents, recorded at different Ca^2+^ concentration. Data were normalised to the current at +50 mV, in 200 μM cytosolic calcium. The continuous lines represented the fitting of the data with the following equation: I = N P_O_(V, [Ca^2+^]_cyt_) V/(1 + ([Ca^2+^]_cyt_/K_OP_) exp(2δFV/RT)). The normalisation factor N, the electrical distance δ and the apparent affinity constant for cytosolic calcium K_OP_ were the free parameters, since the open probability PO was completely determined by the values shown in Supplemental Appendix. A Global fit of the data was not satisfactory, see Supplemental Fig. 2c. Therefore, each data at different cytosolic calcium concentration was fitted separately giving at [Ca^2+^]_cyt_ = 20 μM: δ = 0.34 and K_OP_ = 270 μM; at [Ca^2+^]_cyt_ = 200 μM: δ = 0.44 and K_OP_ = 610 μM. In the text the average of the two values was reported. The normalisation factor was N = 60.9.

We wondered if cytosolic Ca^2+^ could affect the functionality of hTPC1. We therefore performed experiments adding Ca^2+^ in the cytosolic bath solution from nominally 0 up to 200 μM. Very interestingly, as shown in Fig. 4a, currents increased upon an increase of Ca^2+^ from 0 to 20 μM. Normalized conductance plotted against applied voltage demonstrated that raising the cytosolic Ca^2+^ shifted the open probability of the channel toward negative voltages (Fig. 4b). The continuous lines in Fig. 4b were obtained by fitting G_norm_ with a Boltzmann equation: the half activation voltage versus cytosolic Ca^2+^ concentration varied by about 50 mV and saturated at cytosolic Ca^2+^ near 20 μM (Fig. 4c). On the contrary, the slope was not significantly affected by Ca^2+^ (Fig. 4d). By fitting the time course of current activation and deactivation with a single exponential function (see Supplemental Fig. 2a) we found that the effect of [Ca^2+^]_cyt_ on the relaxation time constants was pronounced at positive voltages (Fig. 4e), i.e. the channel activated more rapidly at higher [Ca^2+^]_cyt_.

The [Ca^2+^]_cyt_ dependence of the open probability and of the time constants could be described by the mathematical model shown in Fig. 4f: where C_0_ and C_1_ represent the closed states of the channel respectively without and with a Ca^2+^ ion bound, O_0_ and O_1_ are the open, conductive states without and with Ca^2+^. Transitions between Ca^2+^ bound and Ca^2+^ free states are supposed to be fast, with apparent voltage-independent dissociation constants K_C_ and K_O_; α_i_ and β_i_ are the voltage-dependent rate constants for Ca^2+^ free (i = 0) and Ca^2+^ bound (i = 1) channels, respectively. Details of this four-state model are presented in the Supplemental Appendix. Continuous lines in Fig. 4c,d,e and in Supplemental Fig. 2b, obtained by a global fitting procedure, are in good agreement with the experimental data.

Besides the shift of the open probability towards negative voltages, cytosolic Ca^2+^ increase also induced a voltage-dependent inhibition of the hTPC1 current, as shown in Fig. 4g. The biophysical interpretation of these data suggests that there is at least one site along the permeation pathway that favours Ca^2+^ more than Na^+^ binding. We again used a Woodhull approach^40^, and estimated that this Ca^2+^ binding site was at an electrical distance of 0.39 from the cytosolic side and had an affinity for cytosolic calcium of 440 μM at 0 mV (continuous lines in Fig. 4h; see also Supplemental Fig. 2c).

### Luminal calcium ions have a strong modulatory effect on hTPC1 channel

**Figure 5:**
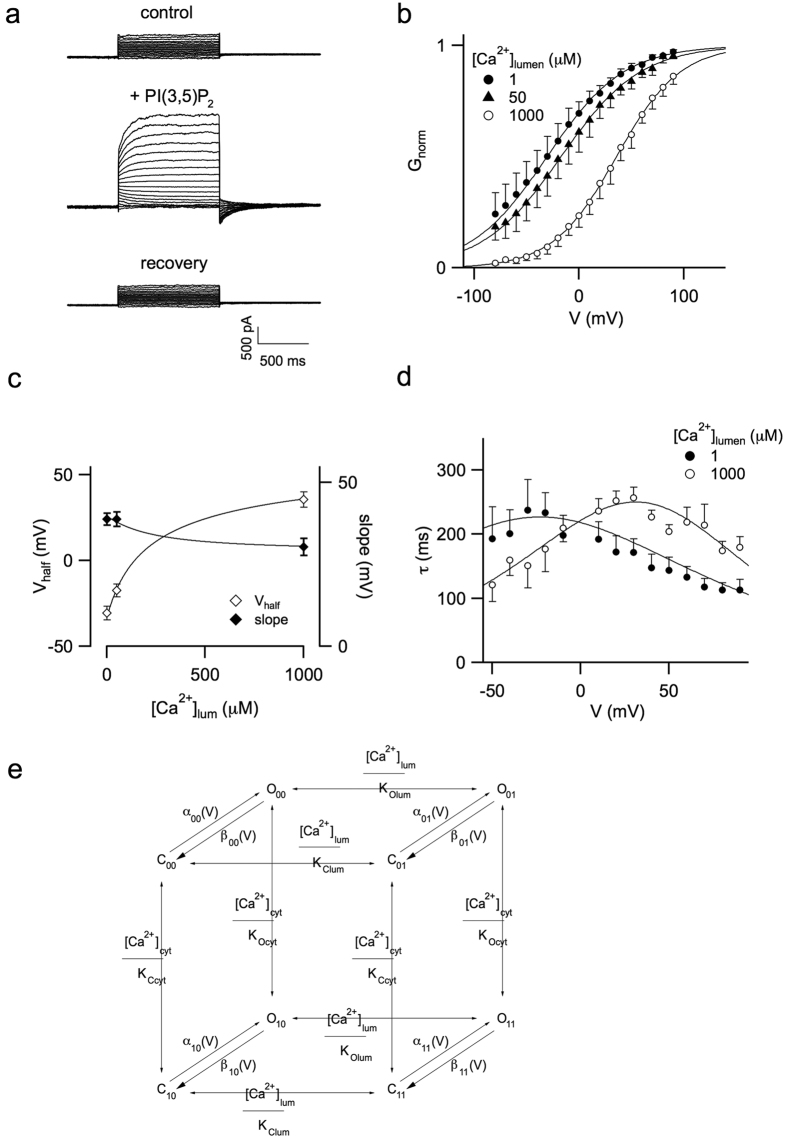
Modulation of hTPC1 by luminal calcium. (**a**) Current recordings obtained before (*top*), during (*middle*) and after (*bottom*) perfusion of 90 nM PI(3,5)P_2_. Main voltage pulse from −80 to +90 mV, step +10 mV; holding voltage: −70 mV, tail voltage at −50 mV. Internal pipette (luminal side) filled with standard solution plus 2 mM EGTA resulting in a free calcium concentration of 1 μM. (**b**) Normalized conductances vs voltage at different Ca^2+^ concentration in the pipette. Data were obtained by the starting value of tail currents at −50 mV. Applied main voltage pulse from −80 to +90 mV. Continuous lines represented fitting of the data by a Boltzmann equation. Data from 4 different vacuoles for each luminal calcium concentration. (**c**) Half activation voltage and slope from the Boltzmann fit of B vs luminal free calcium concentration. (**d**) Relaxation time constants versus voltage at different luminal calcium concentration. For the sake of clarity, data at 50 μM luminal calcium were omitted. They were reported in Supplemental Fig. 3. Continuous lines in c and d were obtained from a global fit of data in b and d by the four state model shown in Supplemental Fig. 3 and described in Supplemental Appendix. (**e**) allosteric mathematical model for hTPC1 modulation by both cytosolic and luminal Ca^2+^.

Luminal Ca^2+^ also had a profound influence on the activity of hTPC1-mediated currents. We performed experiments lowering the Ca^2+^ concentration in the pipette (luminal) solution up to 1 μM by the addition of 2 mM EGTA. Figure 5a show currents recorded without (control and recovery) and adding PI(3,5)P2 in the bath solution. Normalized conductance vs voltage, together with Boltzmann fittings (continuous lines), are displayed in Fig. 5b. The half activation voltage obtained under different luminal Ca^2+^ concentrations in Fig. 5c clearly indicated a large shift (about 60 mV) of the open probability of hTPC1 to positive values, with a parallel slight increase of the slope value, upon luminal Ca^2+^ increase from 1 μM to 1 mM. A similar shift was evident in the relaxation time constants (Fig. 5c). A similar mathematical scheme as developed for the activating effect of cytosolic Ca^2+^ (see scheme of Fig. 4f, Supplemental Appendix and Supplemental Fig. 3a) could nicely describe the inhibition exerted by luminal Ca^2+^ (see continuous lines in Fig. 5c,d and in Supplemental Fig. 3b,c).

### An allosteric model describing modulation of TPC1 by cytosolic and luminal calcium

Our data suggested that hTPC1 channel activation can be finely tuned by Ca^2+^ concentration changes in both cytosolic and luminal compartments. The model presented in Fig. 5e, an extension of the model shown Fig. 4f, summarizes our experimental findings: C and O are respectively the closed and open state of the channel. The two indexes indicate cytosolic and luminal Ca^2+^ binding, respectively; as an example, C_10_ represents the closed state with a cytosolic Ca^2+^ bound and the free site for luminal calcium. Transitions involving cytosolic and luminal Ca^2+^ association and dissociation are hypothesized to be fast compared to opening and closing transitions and voltage-independent. The opening/closing transitions are described by voltage-dependent rate constants, α_ii_ and β_ii_ respectively. K_Ccyt_ and K_Ocyt_, the apparent affinity constants for cytosolic calcium binding to the closed and open state, are 7 and 1 μM, respectively, describing the observation that Ca^2+^ binding favors the open state. Conversely, K_Clum_ and K_Olum_, the apparent affinity binding constants for luminal calcium, are 90 μM and larger than 1 mM, respectively, i.e. luminal Ca^2+^ binding stabilizes the closed state. These values are compatible with the physiological range of cytosolic and luminal Ca^2+^ concentrations (see Discussion). Details of this model and data fittings are presented respectively in the Supplemental Appendix and Supplemental Fig. 4.

## Discussion

In this work we examined in depth the functional characteristics of human TPC1 by using a novel heterologous system. First, we succeeded in expressing the channel in isolated vacuoles from mesophyll cells of Arabidopsis lacking the endogenous TPC1. Noteworthy, to obtain a measurable activity of the channel we had to wait at least two days after protoplast transformation. The phosphoinositide PI(3,5)P_2_ was a powerful activator of hTPC1 with an apparent binding constant of about 15 nM, more powerful than for hTPC2, which in similar experimental conditions showed a two-fold larger binding constant^9^. The activation of TPC1 by PI(3,5)P_2_ found here is in qualitative and quantitative agreement with the results of Cang *et al*.^32^. In similar agreement, we found TPC1 activity to be unaffected by NAADP. Evidence from other groups that TPC channel mediated Ca^2+^ release from acidic organelles is stimulated by NAADP has been rationalized by the hypothesis of an accessory NAADP sensitive protein^41^,^42^,^43^. Such an accessory protein is unlikely to be present in the plant systems, which could explain the NAADP insensitivity of TPC1 observed here and that of TPC2 described earlier^9^.

Our work concentrated on the voltage-dependence, ion selectivity and the Ca^2+^ regulation of hTPC1. The channel was found to be voltage dependent, with an apparent gating valence of about 1 elementary charge, similar to results reported by Cang *et al*.^32^. Currents are highly Na^+^ selective with marginal K^+^ permeability. Experiments in bi-ionic conditions showed that Ca^2+^ can permeate through the channel even though its permeability is significantly lower than the one for Na^+^.

In addition the voltage-dependent Ca^2+^ block observed at rather high cytosolic Ca^2+^ indicates that Ca^2+^ ions are able to enter the pore and bind with higher affinity than Na^+^ to a site at an electrical distance of around 0.4, with an apparent affinity constant larger than 200 μM at 0 mV. This binding site must be separated from the luminal end of the pore by a large energetic barrier to impede significant Ca^2+^ flux.

In addition to the intrapore site, we discovered that TPC1 harbours a rather high affinity cytosolic Ca^2+^ binding site whose occupation allosterically activates the channel by shifting its voltage-dependent characteristic towards negative voltages, and by accelerating the activation time course. Saturating at 20 μM of [Ca^2+^]_cyt_, this effect is compatible with physiological concentration changes of cytosolic Ca^2+^; a simple mathematical model is able to quantitatively describe the measured shift together with the kinetic effects.

Finally, we showed functional evidence of a third Ca^2+^ binding site on the luminal side of the channel. In this case the binding of Ca^2+^ induced a decrease of the activity by shifting the open probability to positive voltages, with a ΔV_half_ of about +60 mV when [Ca^2+^]_lum_ was changed from 1 μM to 1 mM. This might be physiologically relevant since the average luminal concentration of Ca^2+^ has been reported to be around 500 μM and it may vary from 1 mM to less than 1 μM for example upon experimental manipulation of lysosomal pH^44^. Our mathematical model can be nicely extended to include both the cytosolic as well as the luminal Ca^2+^ effect.

Recently the structure of the Arabidopsis plant counterpart of hTPC1, namely AtTPC1, was published^6^,^7^. Interestingly, like hTPC1, AtTPC1 is activated by cytosolic calcium^39^,^45^. Calcium binding is mediated by two EF-hands in the cytosolic loop linking the two monomers^46^. Since hTPC1 has no EF-hands, the mechanism of calcium regulation in the plant channel appears to be very different from that of human TPC1. Similarly, luminal calcium is also decreasing the activity of AtTPC1 by binding to an aspartate residue (D454) located in a luminal loop between transmembrane domains S7 and S8^47^. However, this amino acid is not conserved in hTPC1. Thus, the molecular basis of hTPC1 calcium modulation will require further investigation. On the contrary, the selectivity filter between the plant and the human TPC channels is enough conserved^48^. A recent work^48^ identified two key residues for Na^+^-selectivity in the second pore loop and confirmed that human TPC channels are sodium channel with low calcium permeability.

Overall, our data show that calcium permeation mediated by hTPC1 channels provides a positive-feedback mechanism amplified by both the simultaneous reduction of luminal [Ca^2+^ ] and an increase of cytosolic [Ca^2+^]. In this context Na^+^ is actually a negative regulator since its release from the luminal to cytosolic side depolarizes the luminal membrane and limits Ca^2+^ release. We can speculate that this interplay between Ca^2+^ and Na^+^ is necessary to generate a precise shape and dynamic of Ca^2+^ release or, in other words, a defined Ca^2+^ signature. Our conclusion is therefore that hTPC1 is a Na^+^ and slightly Ca^2+^ permeable, outwardly rectifying channel, working as a sensor of luminal and cytosolic Ca^2+^.

## Materials and Methods

### Plant material and protoplast transformation with human TPC1 sequence

The *hTPC1* coding sequence was PCR amplified from HEK293 (human embryonic kidney) cells cDNA with the Thermo Scientific^®^ Phusion high fidelity polymerase using the following primers: 5′-CATGAAGCTTCATGGCTGTGAGTTTGGATGACGA-3′ and 5′-CATGGAATTCCGCGGTAACGGTCTGGGAGCGCTGG-3′ (underlined are HindIII and EcoRI restriction sites used for cloning).

The PCR product was subsequently digested and ligated into the plant expression vector pSAT6-EGFP-N1 in the Multiple Cloning Site between the HindIII and EcoRI sites downstream of the 35SS strong constitutive promoter in frame with an enhanced GFP. The construct was verified by sequencing and identified as the human TPC1 which is the two pore calcium channel protein 1 isoform X1 coded by XM_011538492.1:246.2696 (mRNA transcript variant X4) that encodes for one of the shorter TPC1 isoforms (816 amino acids).

Plants of *Arabidopsis thaliana tpc1-2*^39^ mutant were grown on soil in a growth chamber at 22 °C and 8 h light/16 h dark regime. Mesophyll protoplasts were isolated from well expanded leaves from four weeks old plants and transiently transformed using the polyethylene glycol method^33^. Protoplasts were maintained up to 5 days at 23°degrees in the dark in W5 solution (in mM, 125 CaCl_2_, 154 NaCl, 5 KCl, 2 MES-KOH, 5 glucose, pH 5.6, ampicillin 100 μg/ml). The transformation efficiency judged by Enhanced Green Fluorescent Protein (EGFP) fluorescence in protoplasts was routinely >50%. An efficient vacuole release was achieved by perfusion of transformed protoplasts with vacuole release solution (in mM: 100 malic acid, 160 1,3-bis(tris(hydroxymethyl)methylamino) propane (BTP), 5 ethylene glycol-bis(2-aminoethylether)-N,N,N′,N′-tetraacetic acid (EGTA), 3 MgCl_2_, pH 7.5, 450 mOsm with D-sorbitol).

### Patch-clamp recordings

Patch-clamp experiments on Arabidopsis vacuoles were performed ≥48 h after protoplast transformation, as described elsewhere^49^,^50^,^51^. To achieve patch-clamp measurements in the whole-vacuole configuration, transformed protoplasts were placed in the recording chamber and exposed to vacuolar release solution (VRS, in mM: 100 malic acid, 160 BTP, 5 EGTA, 3 MgCl_2_, pH = 7.5). TPC-expressing vacuoles were identified by EGFP fluorescence.

The standard pipette (luminal side) solution contained (in mM): 100 NaCl, 2 MgCl_2_, 10 MES, pH 5.5 (with NaOH). The standard bath (cytoplasmic side) solution contained (in mM): 100 NaCl, 10 Hepes, pH 7.5 (NaOH). In the selectivity experiments of Fig. 2, MgCl_2_ 2 mM was added in the cytosolic solution. NaCl 10 mM was obtained by substituting 90 mM of NaCl with equimolar concentration of KCl. In the K^+^-based bath solution of Fig. 3a,b, 100 mM NaCl was replaced with equimolar KCl. To investigate Ca^2+^-permeability, the luminal concentration of NaCl (100 mM) was substituted by 50 mM CaCl_2_. In this condition we observed that the presence of VRS as bath solution increased the quality of the seals. We measured a liquid junction voltage between the two solutions of 9.9 ± 0.6 mV and corrected offline (V = V_applied_ − 9.9 mV). For the sake of clarity in Fig. 3d and Supplemental Fig. 1b,d, the voltage was approximated to the nearest integer. When calcium was not added in the ionic solutions, Ca^2+^ concentration was determined by Plasma Emission Spectrometry (ICP-OES), instrument model Vista PRO Varian (Springvale, Australia), with the following main operating conditions: RF Power: 1100 W; Plasma gas flow rate: 15.0 L min^−1^; Sample uptake rate: 0.8 mL min^−1^. Free calcium concentration in the presence of EGTA was calculated by using a dedicated program^52^. The osmolarity of the luminal and cytoplasmic solutions was adjusted to 550 mOsm and 600 mOsm, respectively, by the addition of D-sorbitol. Dithiothreitol (DTT; 2 mM) was added to the bath solution prior to the measurements^53^. DTT was prepared as 1 M stock solution the day of the experiment and stored in ice. PI(3,5)P_2_ was purchased as dioctanyl ester (diC8) from AG Scientific or Echelon Biosciences Inc (USA). Other chemicals were purchased from Sigma-Aldrich (Italy, Germany). PI(3,5)P_2_ and NAADP were prepared as 0.9 mM and 1 mM stock solutions respectively and stored at −20°.

A total number of more than 95 vacuoles expressing hTPC1 (responding to PI(3,5)P2) were investigated in this work.

### Data analysis

Positive currents correspond to cations flowing from the cytoplasmic side of the vacuole to the lumen or anions moving in the opposite direction. Unless otherwise indicated, data are reported as mean ± sem. For the voltage dependent inhibition mediated by cytosolic calcium, we used the Woodhull model^40^. This approach assumes that the voltage dependence of the effect is due to the existence of a binding site for the ion along the electric field across the membrane. In terms of the model, the exponential voltage-dependence allows to determine the “electrical” distance of the binding site from the membrane surface. Data analysis and figure preparation were done with IgorPro software (Wavemetrics, Lake Oswego, OR, USA).

## Additional Information

**How to cite this article:** Lagostena, L. *et al*. The human two-pore channel 1 is modulated by cytosolic and luminal calcium. *Sci. Rep.* **7**, 43900; doi: 10.1038/srep43900 (2017).

**Publisher's note:** Springer Nature remains neutral with regard to jurisdictional claims in published maps and institutional affiliations.

## Supplementary Material

Supplementary Figures and Appendix
