# Supplementary material for: The human two-pore channel 1 is modulated by cytosolic and luminal calcium
Source: Sci Rep. 2017 Mar 2;7:43900. doi: 10.1038/srep43900 (PMC5333365; doi:10.1038/srep43900)
Supplement: Supplementary Figures and Appendix [file srep43900-s1.pdf]

The human two-pore channel 1 is modulated by cytosolic and luminal calcium

Laura Lagostena, Margherita Festa, Michael Pusch and Armando Carpaneto

Supplemental Fig. 1: Dependence of the reversal voltage by the tail protocol in bi-ionic condition (50 mM $[Ca^{2+}]l_{um}$ / 100 mM $[Na^{+}]_{cyt}$)

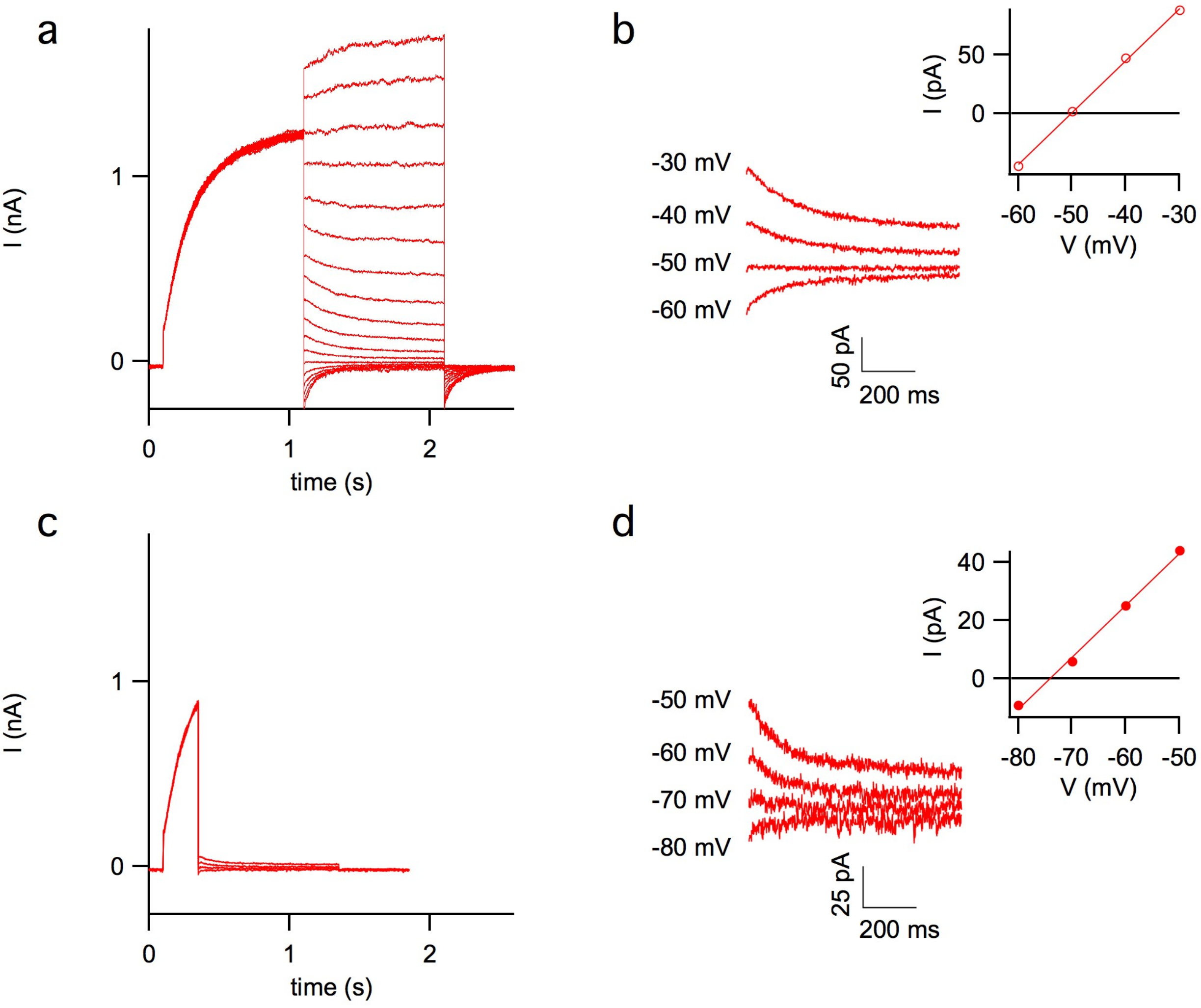

a, Tail currents recorded in 50 mM luminal calcium and 100 mM cytosolic sodium. The main pulse, lasting 1 s, was at +50 mV. Tail voltages of 1 s varied from +70 mV to -100 mV, voltage step of +10 mV and 10 s interval.

b, Magnification of selected tail currents of a. Each tail was fitted by the exponential function $a_0 + a_1 \exp((t-t_0)/\tau)$, where t_0 was the time of the voltage change between the main pulse and the tail (in this condition the delay of about 3 ms introduced by the low-pass, anti-aliasing filter was negligible). By plotting parameter a_1 versus the tail voltage, see inset, and fitting with a linear equation, continuous line in the inset, we could estimate in this experiment a reversal voltage of -50.0 mV and a permeability ratio between calcium and sodium of 0.114, calculated by using the following equation (Lewis CA, 1979. Ion-concentration dependence of the reversal potential and the single channel conductance of ion channels at the frog neuromuscular junction. J Physiol 286:417–445): $\frac{P_{Ca}}{P_{Na}} = \frac{[Na^+]_{acyt}\, e^{\frac{FV_{rev}}{RT}}}{4[Ca^{2+}]_{alum}}(1 + e^{\frac{FV_{rev}}{RT}})$, where $[Na^{+}]_{acyt}$ and $[Ca^{2+}]_{alum}$ are the activities of cytosolic sodium and luminal calcium, respectively, V_{rev} the measured reversal voltage and F, R and T have the usual meaning. The activities were obtained by multiplying the concentration for the

activity coefficient (γ) calculated with the following equation: $\gamma = 10^{\frac{-0.507|z_+ z_-|\sqrt{I}}{1+\sqrt{I}}}$, where z_+ is the valence of sodium (+1) or calcium (+2) respectively, z_- the valence of chloride (-1), I the ionic strength of the solution.
c, A tail protocol with a shorter main pulse was applied to the same vacuole as in A. The main pulse, at 50 mV, was 250 ms long. Tail currents were from -50 mV up to -80, step -10 mV.
d, Magnification of the tail currents shown in c. In the inset, the peak of the exponential function used for fitting the tail currents was plotted against the tail voltage. From the linear approximation of the data, continuous line, we obtained a reversal voltage of -74.0 mV (in this case this determination was more critical than in A because of the small amplitude of the hTPC1 mediated tail currents) and a permeability ratio between calcium and sodium of 0.041. We made the hypothesis that the difference in permeability ratios between A (P_{Ca}/P_{Na}=0.114) and c (P_{Ca}/P_{Na}=0.041) was due to the significant sodium influx occurring during the voltage protocol of a. During a voltage pulse the total positive charge that entered the vacuole was $q = \int_0^{\Delta T} i_p dt$, where ΔT is the duration of the pulse and i_p the positive current. The charge due to the influx of sodium mediated by hTPC1 was $q_{Na} = q - q_{leak}$, where $q_{leak} = i_{leak}\Delta T$. The leak current, i_{leak}, was extrapolated by a linear fitting of the stationary currents elicited at negative voltages under which hTPC1 was closed. We estimated the total charge of sodium that was inside the vacuole at time t_f, just before the reversal voltage was measured, with the following equation: $Q_{Na} = \sum_{i=1}^{n} q_{Na}(i) e^{-\frac{t_f - t_i}{\tau}}$, where i=1, 2, …, n and t_i indicate respectively the number and the initial time of the voltage pulse. The time constant, τ, is linked to the diffusional exchange between the vacuole and the patch pipette (Oliva C, Cohen IS, Mathias RT, 1988. Calculation of time constants for intracellular diffusion in whole cell patch clamp configuration. Biophys J 54:791–799) and is equal to $\tau = \frac{Vol R_a}{D\rho}$. Vol is the volume of the vacuole, R_a the access resistance of the patch-pipette, D = 1.33 10^{-9} $m^2 s^{-1}$ the diffusion coefficient of sodium and ρ = 1.23 Ωm the resistivity of the pipette-filling solution. The volume of the vacuole was calculated by using the following relationship: $Vol = \frac{1}{3\sqrt{4\pi}}\left(\frac{C_m}{C_s}\right)^{\frac{3}{2}}$, where C_s, the specific capacitance of the tonoplast, is assumed to be 10^{-2} pF/μm^2 (Hille B, 2001. Ion Channels of Excitable Membranes, Third Edition, 3rd Edition edition. Sinauer Associates, Sunderland, Mass) R_a and C_m, the total capacitance of the vacuole, were determined by the compensation circuitry of the patch-clamp amplifier or, in alternative, by fitting the transient current induced by a 10 mV voltage step. For the estimation of Q_{Na} we usually considered the voltage pulses applied at least 2τ before the voltage reversal determination. In the vacuole considered here we obtained: R_a=9.9 MΩ, C_m=25.4 pF; therefore τ=73.8 s and the concentration of luminal sodium (Q_{Na}/Vol) in panel a,b was 7.9 mM. The permeability ratio between calcium and sodium was calculated by an extension of the previous equation where the activity of luminal sodium ($[Na^+]_{alum}$) was considered: $\frac{P_{Ca}}{P_{Na}} = \frac{[Na^+]_{acyt} e^{\frac{FV_{rev}}{RT}} - [Na^+]_{alum}}{4[Ca^{2+}]_{alum}}\left(1 + e^{\frac{FV_{rev}}{RT}}\right)$. We obtained a value of 0.051 (the value without correction was 0.114) near to the value of 0.041 determined by the fast tail protocol of c. Therefore this procedure was applied systematically to the data presented in Fig. 3e.

Supplemental Fig. 2: Modelling the cytosolic calcium effects.

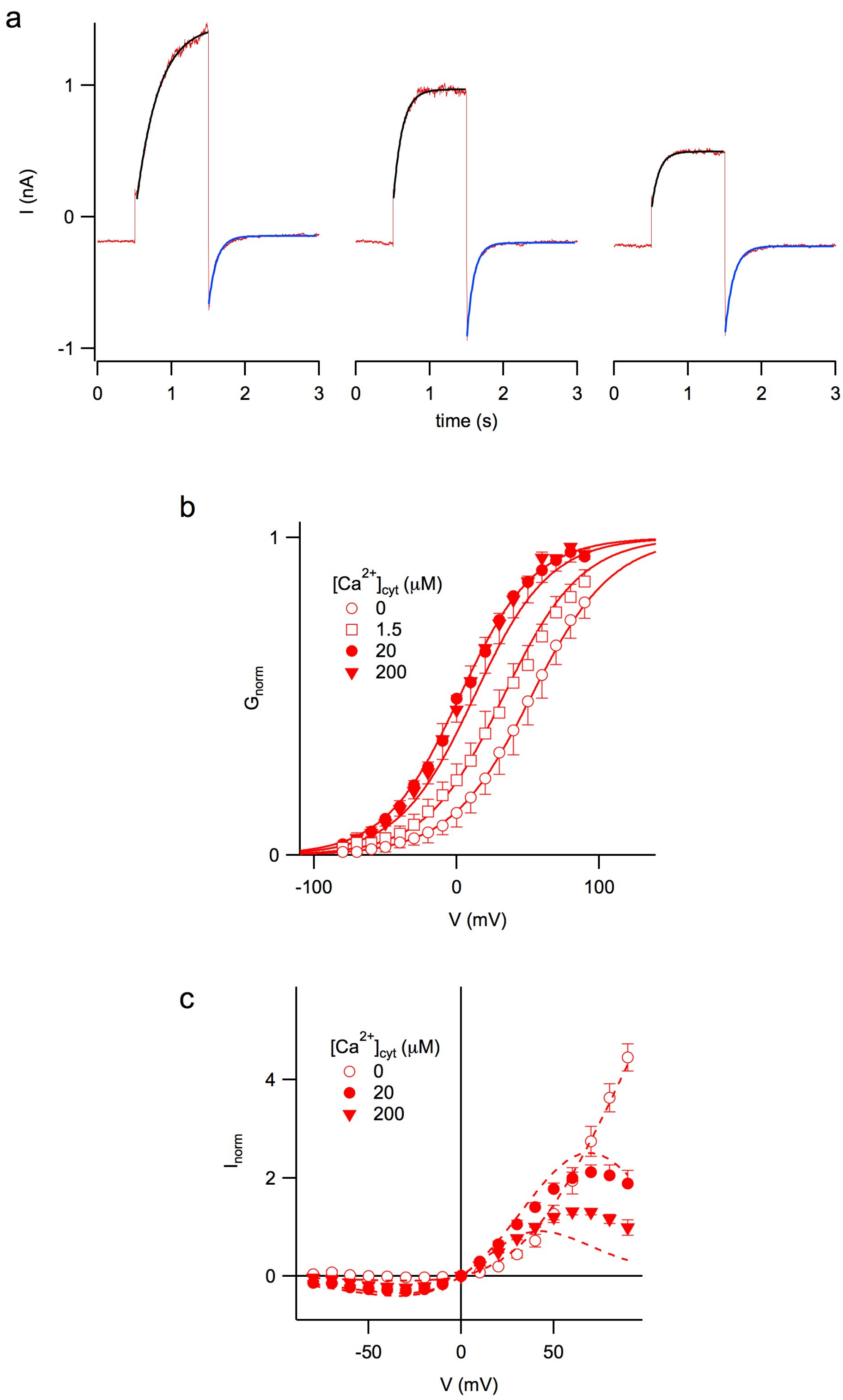

a, Activation and deactivation of hTPC1 mediated currents recorded in different cytosolic calcium concentration (left, middle and right respectively 0, 20 and 200 mM) were fitted by single exponential functions (black and blue thicker lines respectively for activation and deactivation). Activation and deactivation voltages at +90 and -50 mV, respectively.

b, G_{norm}, the normalized conductance obtained from tail current peaks at -50 mV, was plotted as a function of the main voltage pulse at different cytosolic Ca^{2+} concentrations. Continuous lines were obtained with a global fitting procedure by using the equations derived by scheme of Fig. 4f (see text and Supplemental Appendix for mathematical details and numerical solutions).
c, data as in Fig. 4g; the dashed lines represented the global fit of the data with a Woodhull model (see text of Fig. 4g legend).

Supplemental Fig. 3: Modelling the luminal calcium effect.

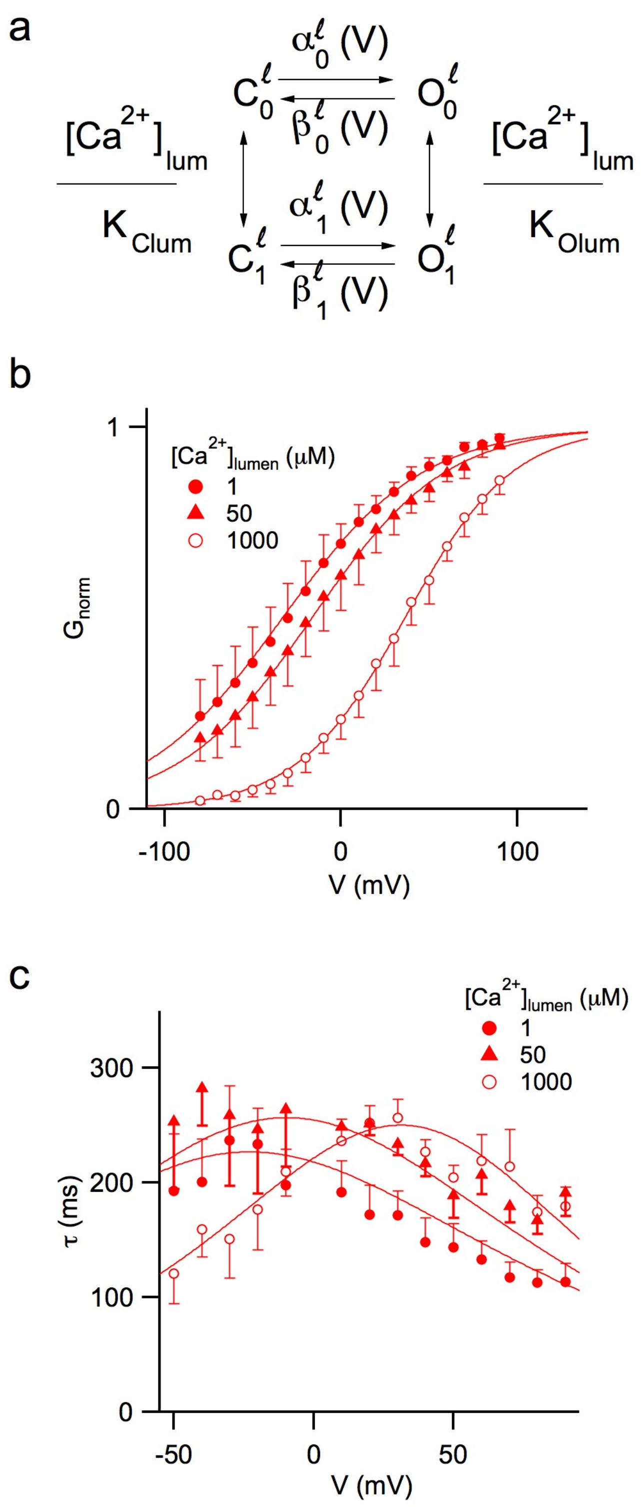

a, mathematical model used to describe the effect of luminal calcium change. The mathematical description of the model, the same of scheme of Fig. 4f, is reported in the Supplemental Appendix.
b, same data as in Fig. 5b. Continuous lines were obtained with a global fitting procedure by using the model of a.
c, Relaxation time constants versus voltages as in Fig. 5d. Data recorded in 50 μM luminal calcium were added.

Supplemental Fig. 4: The eight-state model.

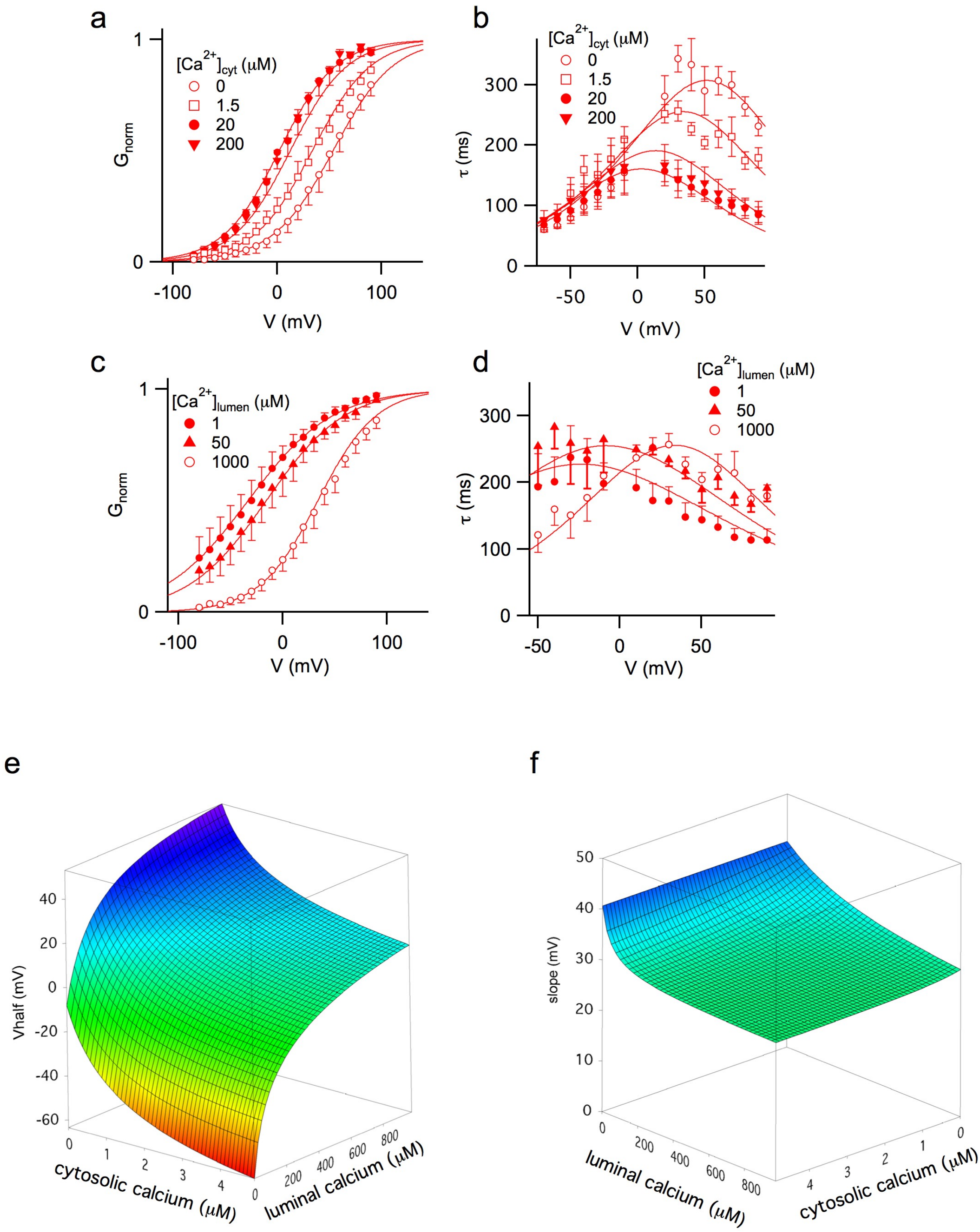

a, b, c and d: data as respectively in Fig. 4b, 4e, 5b, Supplemental Fig. 3c. The continuous lines were obtained by scheme of Fig. 5e with a global fitting procedure. Mathematical details and numerical results are reported in Supplemental Appendix.

e, f, Surface plot of respectively Vhalf and slope obtained by scheme of Fig. 5e versus cytosolic and luminal calcium. Details are presented in Supplemental App.

Supplemental Appendix: mathematical description of the models

Modulation by cytosolic calcium: scheme of Fig. 4f

Under the hypothesis of fast equilibrium between the channel states without and with cytosolic calcium bound, scheme of Fig. 4f could be reduced to this simple two state scheme:

$$C \underset{\beta^*(V)}{\overset{\alpha^*(V)}{\rightleftarrows}} O$$

where

$$\alpha^* = \frac{\alpha_0 + \alpha_1 \frac{[Ca^{2+}]_{cyt}}{K_C}}{1 + \frac{[Ca^{2+}]_{cyt}}{K_C}} \tag{1}$$

and

$$\beta^* = \frac{\beta_0 + \beta_1 \frac{[Ca^{2+}]_{cyt}}{K_O}}{1 + \frac{[Ca^{2+}]_{cyt}}{K_O}} \tag{2}$$

The open probability and the relaxation time constant of the channel, both dependent on voltage and cytosolic calcium were:

$$P_O\left(V, [Ca^{2+}]_{cyt}\right) = \frac{1}{1 + \frac{\beta^*}{\alpha^*}} = \frac{1}{1 + \frac{1 + \frac{[Ca^{2+}]_{cyt}}{K_C}}{1 + \frac{[Ca^{2+}]_{cyt}}{K_O}} \cdot \frac{\beta_0 + \beta_1 \frac{[Ca^{2+}]_{cyt}}{K_O}}{\alpha_0 + \alpha_1 \frac{[Ca^{2+}]_{cyt}}{K_C}}} \tag{3}$$

$$\tau\left(V, [Ca^{2+}]_{cyt}\right) = \frac{1}{\alpha^* + \beta^*} = \frac{1}{\frac{\alpha_0 + \alpha_1 \frac{[Ca^{2+}]_{cyt}}{K_C}}{1 + \frac{[Ca^{2+}]_{cyt}}{K_C}} + \frac{\beta_0 + \beta_1 \frac{[Ca^{2+}]_{cyt}}{K_O}}{1 + \frac{[Ca^{2+}]_{cyt}}{K_O}}}$$

(4)

The single rate constants could be expressed as:

$\alpha_i(V) = \bar{\alpha}_i e^{\frac{z_{\alpha i} F}{RT} V}$ and $\beta_i(V) = \bar{\beta}_i e^{-\frac{z_{\beta i} F}{RT} V}$ with i=0,1. From microscopic reversibility we obtained the following relationship, which linked the voltage independent parameters of the model:

$$\bar{\beta}_1 = \bar{\alpha}_1 \frac{K_O}{K_C} \frac{\bar{\beta}_0}{\bar{\alpha}_0} \tag{5}$$

Equations 3 and 4 were used to perform a global fit of the data presented in Fig. 4b and 4e, respectively. The fittings were the continuous lines shown in Supplemental Fig. 2b and in Fig. 4e. The values of the nine free parameters obtained by the fitting procedure were: $\bar{\alpha}_0$=0.661, $z_{\alpha 0}$=0.436, $\bar{\beta}_0$=4.14, $z_{\beta 0}$=0.448, $\bar{\alpha}_1$=3.07, $z_{\alpha 1}$=0.462, $z_{\beta 1}$=0.501, K_C=6.90 μM, K_O=1.16 μM.

Equation 3, which represented the open probability, was not an exact Boltzmann function. In the figure below the red continuous line, which represented equation 3 at 1.5 μM cytosolic calcium, was fitted by a Boltzmann function (black dashed line).

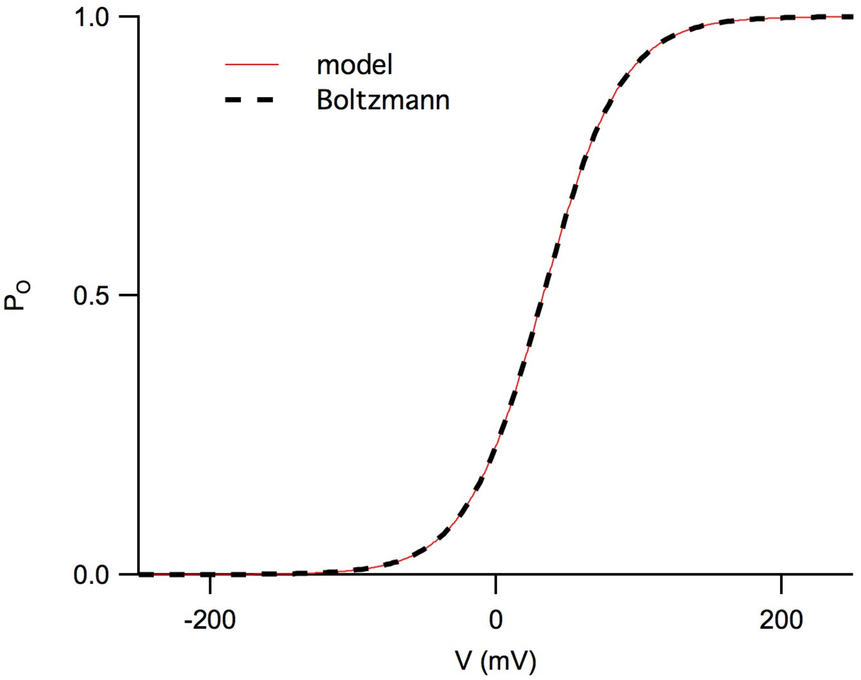

In this example, the agreement between the two curves was very good. We therefore applied the same approach varying cytosolic calcium to find the theoretical V_{half} and slope plotted as continuous lines in Fig. 4c and 4d.

Modulation by luminal calcium

The modulation by luminal calcium could be described by the mathematical model shown in Supplemental Fig. 3a, which was identical to scheme of Fig. 4f. Also in this case we assumed 1) fast equilibrium between the channel state without and with luminal calcium bound; 2) that the affinity constant K_{Clum} and K_{Olum} were voltage-independent. We used equations 3 and 4 to perform a global fit of the data presented respectively in Fig. 5b and Fig. 5d (Supplemental Fig. 3c is the complete version of 5d). The fittings were shown as continuous lines in Supplemental Fig. 3b,c and

in Fig. 5d. The values of the free parameters were: $\bar{\alpha}_0^l$=3.23, $z_{\alpha 0}^l$=0.276, $\bar{\beta}_0^l$=1.38, $z_{\beta 0}^l$=0.360, $\bar{\alpha}_1^l$=0.870, $z_{\alpha 1}^l$=0.490, $z_{\beta 1}^l$=0.387, K_{Clum}=90 μM, K_{Olum}=1.68 mM. The theoretical V_{half} and slope shown as continuous lines in Fig. 5c were obtained as described above.

The eight-state model: scheme of Fig. 5e

Scheme of Fig. 5e was an extension of scheme of Fig. 4f. Under our hypothesis, see text, the model could be reduced to the two-state system with the following rate constants:

$$\alpha^* = \frac{\alpha_{00}+\alpha_{10}\frac{[Ca^{2+}]_{cyt}}{K_{Ccyt}}+\alpha_{01}\frac{[Ca^{2+}]_{lum}}{K_{Clum}}+\alpha_{11}\frac{[Ca^{2+}]_{cyt}}{K_{Ccyt}}\cdot\frac{[Ca^{2+}]_{lum}}{K_{Clum}}}{\left(1+\frac{[Ca^{2+}]_{cyt}}{K_{Ccyt}}\right)\left(1+\frac{[Ca^{2+}]_{lum}}{K_{Clum}}\right)} \quad (6)$$

$$\beta^* = \frac{\beta_{00}+\beta_{10}\frac{[Ca^{2+}]_{cyt}}{K_{Ocyt}}+\beta_{01}\frac{[Ca^{2+}]_{lum}}{K_{Olum}}+\beta_{11}\frac{[Ca^{2+}]_{cyt}}{K_{Ocyt}}\cdot\frac{[Ca^{2+}]_{lum}}{K_{Olum}}}{\left(1+\frac{[Ca^{2+}]_{cyt}}{K_{Ocyt}}\right)\left(1+\frac{[Ca^{2+}]_{lum}}{K_{Olum}}\right)} \quad (7)$$

The open probability and the relaxation time constant were:

$$P_O(V,[Ca^{2+}]_{cyt},[Ca^{2+}]_{lum}) = \frac{1}{1+\frac{\beta^*}{\alpha^*}} =$$

$$= \frac{1}{1+\frac{\left(1+\frac{[Ca^{2+}]_{cyt}}{K_{Ccyt}}\right)\left(1+\frac{[Ca^{2+}]_{lum}}{K_{Clum}}\right)}{\left(1+\frac{[Ca^{2+}]_{cyt}}{K_{Ocyt}}\right)\left(1+\frac{[Ca^{2+}]_{lum}}{K_{Olum}}\right)}\cdot\frac{\beta_{00}+\beta_{10}\frac{[Ca^{2+}]_{cyt}}{K_{Ocyt}}+\beta_{01}\frac{[Ca^{2+}]_{lum}}{K_{Olum}}+\beta_{11}\frac{[Ca^{2+}]_{cyt}}{K_{Ocyt}}\cdot\frac{[Ca^{2+}]_{lum}}{K_{Olum}}}{\alpha_{00}+\alpha_{10}\frac{[Ca^{2+}]_{cyt}}{K_{Ccyt}}+\alpha_{01}\frac{[Ca^{2+}]_{lum}}{K_{Clum}}+\alpha_{11}\frac{[Ca^{2+}]_{cyt}}{K_{Ccyt}}\cdot\frac{[Ca^{2+}]_{lum}}{K_{Clum}}}} \quad (8)$$

$$\tau(V,[Ca^{2+}]_{cyt},[Ca^{2+}]_{lum}) = \frac{1}{\alpha^*+\beta^*} =$$

$$= \frac{1}{\frac{\alpha_{00}+\alpha_{10}\frac{[Ca^{2+}]_{cyt}}{K_{Ccyt}}+\alpha_{01}\frac{[Ca^{2+}]_{lum}}{K_{Clum}}+\alpha_{11}\frac{[Ca^{2+}]_{cyt}}{K_{Ccyt}}\cdot\frac{[Ca^{2+}]_{lum}}{K_{Clum}}}{\left(1+\frac{[Ca^{2+}]_{cyt}}{K_{Ccyt}}\right)\left(1+\frac{[Ca^{2+}]_{lum}}{K_{Clum}}\right)}+\frac{\beta_{00}+\beta_{10}\frac{[Ca^{2+}]_{cyt}}{K_{Ocyt}}+\beta_{01}\frac{[Ca^{2+}]_{lum}}{K_{Olum}}+\beta_{11}\frac{[Ca^{2+}]_{cyt}}{K_{Ocyt}}\cdot\frac{[Ca^{2+}]_{lum}}{K_{Olum}}}{\left(1+\frac{[Ca^{2+}]_{cyt}}{K_{Ocyt}}\right)\left(1+\frac{[Ca^{2+}]_{lum}}{K_{Olum}}\right)}} \quad (9)$$

The single rate constants were $\alpha_{ii}(V) = \bar{\alpha}_{ii}e^{\frac{z_{\alpha ii}F}{RT}V}$ and $\beta_{ii}(V) = \bar{\beta}_{ii}e^{-\frac{z_{\beta ii}F}{RT}V}$ with i=0,1. From microscopic reversibility the following relationships were obtained:

$$\bar{\beta}_{10} = \bar{\alpha}_{10}\frac{K_{Ocyt}}{K_{Ccyt}}\frac{\bar{\beta}_{00}}{\bar{\alpha}_{00}} \quad (10)$$

$$\bar{\beta}_{01} = \bar{\alpha}_{01} \frac{K_{Olum}}{K_{Clum}} \frac{\bar{\beta}_{00}}{\bar{\alpha}_{00}} \quad (11)$$

$$\bar{\beta}_{11} = \bar{\alpha}_{11} \frac{K_{Ocyt}}{K_{Ccyt}} \frac{K_{Olum}}{K_{Clum}} \frac{\bar{\beta}_{00}}{\bar{\alpha}_{00}} \quad (12)$$

Equations 8 and 9 were used to perform a global fit of the data presented in Fig. 4b,e and 5b, d (Supplemental Fig. 3c is the complete version of 5d) with affinity constants for cytosolic and luminal calcium fixed to the values determined in the previous paragraphs (i.e.: K_{Ccyt}=6.90 μM, K_{Ocyt}=1.16 μM, K_{Clum}=90 μM, K_{Olum}=1.68 mM). The other free 13 free parameters were found by the fitting procedure and were: $\bar{\alpha}_{00}$ =3.87, $z_{\alpha 00}$ =0.272, $\bar{\alpha}_{10}$ =0.201, $z_{\alpha 10}$ =0.442, $\bar{\alpha}_{01}$ =0.360, $z_{\alpha 01}$ =0.551, $\bar{\alpha}_{11}$=3.35, $z_{\alpha 11}$=0.460, $\bar{\beta}_{00}$=3.17, $z_{\beta 00}$=0.350, $z_{\beta 10}$=0.174, $z_{\beta 01}$=0.518, $z_{\beta 11}$=0.477. The fittings were shown as continuous lines in Supplemental Fig. 4a,b,c,d. The theoretical V_{half} and slope were determined with a similar procedure as described above and were shown as surface plots versus cytosolic and luminal calcium in Supplemental Fig. 4e,f.
